# Algorithm for Turning Detection and Analysis Validated under Home-Like Conditions in Patients with Parkinson’s Disease and Older Adults using a 6 Degree-of-Freedom Inertial Measurement Unit at the Lower Back

**DOI:** 10.3389/fneur.2017.00135

**Published:** 2017-04-10

**Authors:** Minh H. Pham, Morad Elshehabi, Linda Haertner, Tanja Heger, Markus A. Hobert, Gert S. Faber, Dina Salkovic, Joaquim J. Ferreira, Daniela Berg, Álvaro Sanchez-Ferro, Jaap H. van Dieën, Walter Maetzler

**Affiliations:** ^1^Department of Neurodegeneration, Center for Neurology, Hertie Institute for Clinical Brain Research (HIH), University of Tübingen, Tübingen, Germany; ^2^German Center for Neurodegenerative Diseases, DZNE, Tübingen, Germany; ^3^Department of Neurology, Christian-Albrechts-University, Kiel, Germany; ^4^Department of Human Movement Sciences, MOVE Research Institute Amsterdam, VU University Amsterdam, Amsterdam, Netherlands; ^5^Clinical Pharmacology Unit, Instituto de Medicina Molecular, Lisbon, Portugal; ^6^Laboratory of Clinical Pharmacology and Therapeutics, Faculty of Medicine, University of Lisbon, Lisbon, Portugal; ^7^HM CINAC, Hospital Universitario HM Puerta del Sur, Móstoles, Spain; ^8^Research Laboratory of Electronics, Massachusetts Institute of Technology, Cambridge, MA, USA

**Keywords:** six degrees of freedom, accelerometer, daily activities, gyroscope, older adults, Parkinson’s disease, turning

## Abstract

**Introduction:**

Aging and age-associated disorders such as Parkinson’s disease (PD) are often associated with turning difficulties, which can lead to falls and fractures. Valid assessment of turning and turning deficits specifically in non-standardized environments may foster specific treatment and prevention of consequences.

**Methods:**

Relative orientation, obtained from 3D-accelerometer and 3D-gyroscope data of a sensor worn at the lower back, was used to develop an algorithm for turning detection and qualitative analysis in PD patients and controls in non-standardized environments. The algorithm was validated with a total of 2,304 turns ≥90° extracted from an independent dataset of 20 PD patients during medication ON- and OFF-conditions and 13 older adults. Video observation by two independent clinical observers served as gold standard.

**Results:**

In PD patients under medication OFF, the algorithm detected turns with a sensitivity of 0.92, a specificity of 0.89, and an accuracy of 0.92. During medication ON, values were 0.92, 0.78, and 0.83. In older adults, the algorithm reached validation values of 0.94, 0.89, and 0.92. Turning magnitude (difference, 0.06°; SEM, 0.14°) and duration (difference, 0.004 s; SEM, 0.005 s) yielded high correlation values with gold standard. Overall accuracy for direction of turning was 0.995. Intra class correlation of the clinical observers was 0.92.

**Conclusion:**

This wearable sensor- and relative orientation-based algorithm yields very high agreement with clinical observation for the detection and evaluation of ≥90° turns under non-standardized conditions in PD patients and older adults. It can be suggested for the assessment of turning in daily life.

## Introduction

Turning is a regularly performed movement relevant for the successful performance of daily life activities. It requires multi-limb coordination and continuous change of center of mass (1) and thus provides information about dynamic balance aspects (2). It is, therefore, not surprising that turning deficits are associated with increased risk of falling (3, 4), with consequences such as fractures (5) and increased risk of mortality (6). Turning deficits are commonly observed in older adults (7) and patients with Parkinson’s disease (PD) (8, 9). For example, older adults with difficulties in turning show more staggering during turns, take more steps, and show a longer duration to complete one turn than do young adults. PD patients turn slower and require more steps to complete one turn, compared to young adults. Therefore, valid assessment of this movement and its deficits, in particular in unobserved environments in these cohorts, may have a large potential for the design of specific treatment approaches and for prevention of severe consequences.

Assessment of turning is not trivial. Optical systems, which have been widely used in previous experiments (7, 10–12), are costly and can only be used in the laboratory. Wearable sensors are a promising alternative (13–16). They are relatively small (therefore minimally obstruct normal behavior), cheap, assess unlimited movement areas as they are worn on the body, collect data independently from other sources, and have the ability to measure over days and even weeks. As a consequence, they are ideal data collectors in non-standardized (i.e., unconstrained, natural, home) environments over long periods of time. Algorithms for the analysis of turning movements for young and older adults (17) and PD patients (9) have already been published. However, they suffer from downsides that may hinder a wide use in clinical routine and large studies. A particularly interesting publication with regard to the results presented here (9) analyzed a circuit, walked by 21 PD patients and 19 older adults in the lab, with short straight walks interspersed with 10 turns of 45°, 90°, 135°, and 180° under slow, self-preferred, and fast speeds. During the assessments, participants wore a sensor at the lower back. An algorithm built on nine degrees of freedom (9DOF, which means using data from accelerometer, gyroscope, and magnetometer) fusion extracted turning frequency, which was then compared against video observation and motion capture analysis. The algorithm yielded a sensitivity of 0.75 (rater 1) and 0.77 (rater 2), and a specificity of 0.60/0.69. This previously published algorithm was validated only in standardized conditions, where the path of walking and the number and magnitude of turns have been pre-defined. Thus, the accuracy under free-living like conditions has not been determined in previous work.

Based on these preliminary but promising results, we set out to develop an improved algorithm for turning detection and analysis for PD patients and older adults in a non-standardized environment based on six degrees of freedom (6DOF, using data from accelerometer and gyroscope) signal information, using data extracted from a sensor worn on the lower back. This method was validated in non-standardized free-living-like conditions, during a reasonably long testing period (90 min for controls and 180 min for PD patients). The method also redefined turn hesitations, to cover more discrete turns. Here, we present details of the algorithm and results on its validation.

## Methods

### Study Participants and Settings

The study was performed at the University Hospital Tübingen. Twenty-five patients were recruited from the ward and the outpatient clinic. Fourteen spouses of the patients served as controls. Inclusion criteria were age between 50 and 90 years and, for PD patients, a Hoehn and Yahr (H&Y) score between 1 and 3. Exclusion criteria were deep brain stimulation and a Mini-Mental State Examination score <24. Patients were diagnosed by experienced movement disorders specialists. Controls underwent a neurological examination to exclude presence of neurological symptoms indicative of a neurodegenerative process. Details are provided in Ref. (18). The protocol (nr. 399/2012BO2) was approved by the local ethics committee on 27 September 2012 and was in accordance with the Declaration of Helsinki. All participants gave informed written consent before the assessments.

Duration of the assessment was approximately 90 min per participant and medication condition. PD patients were assessed during medication ON and OFF. Comparable to Ref. (19), medication OFF was defined as overnight withdrawal from dopaminergic medication, and medication ON as the study participant’s perception of having a “Good On Phase,” after regular intake of medication. The assessment consisted of daily activity-like procedures, such as walking in the rooms and corridors of the lab environment without restriction, opening and closing doors, climbing stairs, performing transfers such as sit-to-stand, sit-to-walk, stand-to-sit and walk-to-sit, brushing teeth, making coffee, drinking a cup of tea, and ironing (18). During the assessments, the participants wore a sensor on the lower back (OPAL system, APDM, Inc., Portland, OR, USA). One of the coauthors followed and recorded the objects with a video camera. The camera has a resolution of 1,920 × 1,080 pixels, and a sampling frequency of 50 Hz. The synchronization between sensors and videos was guaranteed with an additional inertial measurement unit (IMU) worn on the ankle, when participants performed specific movements [foot tapping during Unified Parkinson’s Disease Rating Scale (UPDRS) performance]. The IMU at the back and the IMU on the ankle were synchronized as described on the homepage of the IMU provider (APDM) (20). These videotapes were evaluated by two independent clinical observers. For both the algorithm and the clinical observers, a turn was defined as a movement around the vertical axis during standing or walking within 0.1 to 10 s. Clinical observers rated every vertical rotation ≥45° as a turn, while the algorithm rated ≥45°, ≥60°, ≥70°, ≥80°, ≥90°, ≥100°, ≥110° as different turn thresholds. All turns detected during the blind-spot periods (i.e., when the hip of the study participants was out of the camera field) were removed (8% of sensor-detected turns). In addition, the motor part of the UPDRS (UPDRS-III) was administered in PD patients during medication ON and OFF separately (21).

The cohort was then split into a training cohort for algorithm development and a test cohort for algorithm validation. For the iterative algorithm development process [phase A according to Ref. (22)], sensor and videotape data of the above-mentioned daily activity-like procedures from five PD (both ON- and OFF-conditions) patients and one control were included. We selected PD patients with relatively “difficult” conditions, such as dyskinesia and tremor, for the detection of turns, to give the algorithm developer (MHP) the opportunity to iteratively train the algorithm under challenging conditions. Turns of this cohort were also used to define the most useful magnitude threshold of a turn to be implemented in the algorithm for further analyses. For the validation phase [phase B according to Ref. (22)], sensor and clinical observer data of the daily activity-like procedures from the remaining 20 PD patients and 13 controls were included. Detailed demographic and clinical information about the training and test cohorts is provided in Table 1.

**Table 1:** **Demographic and clinical data of the training and test cohorts**.

	PD	Older adults
**Training cohort**
*N* (females)	5 (3)	1 (1)
Age (years)	71 (4)	51
UPDRS III (0–132)	23 (10)	3 (0)
H&Y (0–5)	2 (1)	0 (0)
LED (mg)	713 (640)	(0)
**Test cohort**
*N* (females)	20 (10)	13 (6)
Age (years)	66 (9)	60 (10)
UPDRS III (0–132)	32 (12)	2 (4)
H&Y (0–5)	3 (1)	0 (0)
LED (mg)	839 (622)	0 (0)

*Data are shown as mean ± SD, except gender and observed turns*.

*H&Y, Hoehn & Yahr; LED, Levodopa equivalent dose; PD, Parkinson’s disease; UPDRS, Unified Parkinson’s Disease Rating Scale III, motor part of the revised Unified PD Rating scale*.

### Algorithm Development and Structure

**Figure 1:**
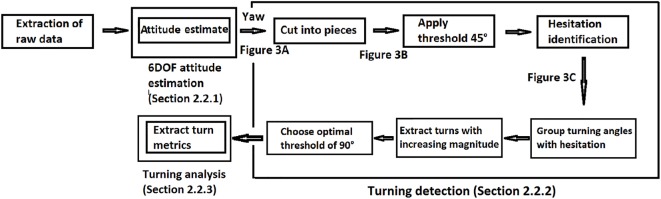
**General structure of the algorithm for turning detection and analysis**. A turn was defined as a yaw angle (angle change around vertical axis) with a magnitude ≥90° and a duration of 0.1–10 s (for details see Section “Methods” Figures 2 and 3).

The development of the algorithm for turning detection and analysis was performed with MATLAB R2015b and consisted of three steps: 6DOF attitude estimation, turning detection, and turning analysis (Figure 1).

#### 6DOF Attitude Estimation Based on Relative Orientation

**Figure 2:**
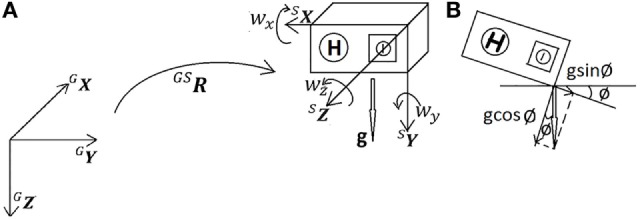
**Six degrees of freedom attitude estimation**. **(A)** Relationship between global frame (G-frame) and sensor frame (S-frame) was described by rotation matrix ^GS^***R***. During stable phases, the accelerometer measures gravity *g*, and the gyroscope measures angular velocity [w*_x_* w*_y_* w*_z_*], in sensor frame S. **(B)** Presence of an inclination angle ø led to a change from vertical axis ^G^***Z*** to ${\;}_{\text{S}}^{\text{G}}\text{Z}$ (gravity *g* was split into cosine and sine terms).

The basic principle of the 6DOF attitude estimation is built on the relative orientation of the sensor with respect to the reference frame (global frame, G-frame), using a rotation matrix ^GS^***R*** (S stands for sensor frame, S-frame). Each frame is considered by definition a triad of the orthogonal vectors ***X***, ***Y***, ***Z*** (Figure 2A). ^GS^***R*** expresses the difference between the G- and S-frame in a 3 × 3 matrix. The definition of ^GS^***R*** will then be ${\left[{\;}_{\text{S}}^{\text{G}}{\text{X}}_{\text{S}}^{\text{G}}{\text{Y}}_{\text{S}}^{\text{G}}\text{Z}\right]}^{\text{T}}$, where ${\;}_{\text{S}}^{\text{G}}{\text{X}}_{\text{S}}^{\text{G}}{\text{Y}}_{\text{S}}^{\text{G}}\text{Z}$ describe the axes of the G-frame expressed in the S-frame.

The inclination of the sensor is described by the angle ø relative to gravity, which is chosen to be our ***Z*** vector (Figure 2B): ^G^***Z*** =[0 0 1]^T^, and ${\;}_{\text{S}}^{\text{G}}\text{Z}=\text{normalize}\;\left(g{\left[\text{sinø cosø}0\right]}^{\text{T}}\right)\;={\left[\text{sinø cosø}\;0\right]}^{\text{T}}$. ${\;}_{\text{S}}^{\text{G}}\text{Z}$ is calculated from the first phase of the measurement when five samples of the accelerometer data (a*_x_* a*_y_* a*_z_*) are stable (in the first 3.9% of a second in case of a sampling rate of 128 Hz, “stable” is defined as peak-to-peak change in accelerometer amplitude $<0.2^{\frac{\text{m}}{{\text{s}}^{2}}}$).

Then, ${\;}_{\text{S}}^{\text{G}}\text{X}$ was randomly chosen as [1 0 0]^T^. The cross product between ${\;}_{\text{S}}^{\text{G}}\text{X}$ and ${\;}_{\text{S}}^{\text{G}}\text{Z}$ gives ${\;}_{\text{S}}^{\text{G}}\text{Y}$. From this result, the cross product between ${\;}_{\text{S}}^{\text{G}}\text{Y}$ and ${\;}_{\text{S}}^{\text{G}}\text{Z}$ was again calculated, to guarantee orthogonality:

$$\begin{array}{llll} {\;}_{\text{S}}^{\text{G}}\text{X} & ={\left[1\;0\;0\right]}^{\text{T}}\; & & \;\\ {\;}_{\text{S}}^{\text{G}}\text{Y} & {=}_{\text{S}}^{\text{G}}\text{Z}\times_{\text{S}}^{\text{G}}\text{X}\; & & \;\\ {\;}_{\text{S}}^{\text{G}}\text{X} & {=}_{\text{S}}^{\text{G}}\text{Y}\times_{\text{S}}^{\text{G}}\text{Z}\; & & \; \end{array}$$

The magnitudes of the respective vectors ${\;}_{\text{S}}^{\text{G}}{\text{X}}_{\text{S}}^{\text{G}}{\text{Y}}_{\text{S}}^{\text{G}}\text{Z}$ were normalized to assure that they have unit length. Then they constitute the initial relative orientation of the sensor, ^GS^***R***.

Changes in orientation were then determined from ^GS^***R*** and ^G^**w**, where ^G^**w** was calculated from the sensor gyroscope output ^S^**w** (=[^S^w*_x_* ^S^w*_y_* ^S^w*_z_*]^T^), based on the formula

$${\;}_{\;}^{\text{G}}\text{w}{=}_{\;}^{\text{GS}}\text{R}{*}_{\;}^{\text{S}}\text{w}.$$

^GS^***R*** is updated every sample (1/f_s_) by the following formula (23)

$${\;}_{\text{new}}^{\text{GS}}\text{R}{=}_{\text{transform}}^{\;\text{GS}}\text{R}{*}_{\text{old}}^{\text{GS}}\text{R}$$

$${\;}_{\text{transform}}^{\;\text{GS}}\text{R}=\left[\begin{array}{ccc} \text{t}*{{\text{w}}_{x}}^{2}+\text{c} & \text{t}*{\text{w}}_{x}*{\text{w}}_{y}+\text{s}*{\text{w}}_{z} & \text{t}*{\text{w}}_{x}*{\text{w}}_{z}-\text{s}*{\text{w}}_{y}\\ \text{t}*{\text{w}}_{x}*{\text{w}}_{y}-\text{s}*{\text{w}}_{z} & \text{t}*{{\text{w}}_{y}}^{2}+\text{c} & \text{t}*{\text{w}}_{y}*{\text{w}}_{z}+\text{s}*{\text{w}}_{x}\\ \text{t}*{\text{w}}_{x}*{\text{w}}_{z}+\text{s}*{\text{w}}_{y} & \text{t}*{\text{w}}_{y}*{\text{w}}_{z}-\text{s}*{\text{w}}_{x} & \text{t}*{{\text{w}}_{z}}^{2}+\text{c} \end{array}\right]$$

with c is cos(θ), s is sin(θ), t is 1 − cos(θ), and θ is the angle of rotation = Norm(**w**)/f_s_. Note that the S before the **w** is not displayed for readability issues.

In a final step of the 6DOF attitude estimation, ^GS^***R*** was converted to Euler angles (roll–pitch–yaw) for the detection of turning, where roll represents the angle displacements around ***X***, pitch around ***Y***, and yaw around ***Z***. Only yaw (i.e., *the angular displacement around the ***Z*** axis*) was considered for the next step (see Turning Detection). Note that with choosing ${\;}_{\text{S}}^{\text{G}}\text{X}$ to be [1 0 0]^T^, yaw was initially chosen as 0.

#### Turning Detection

**Figure 3:**
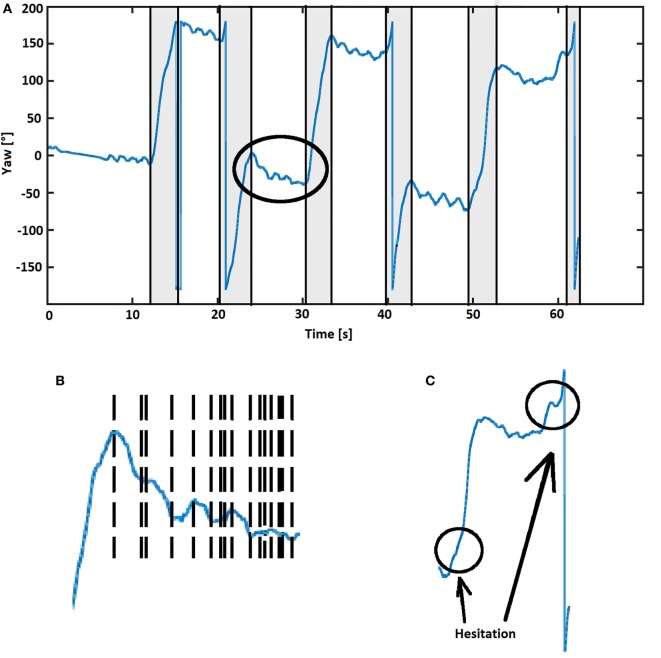
**(A)** Turning pattern example from a test person. Six gray rectangular regions reflect turns detected by the algorithm, with flags at the beginning and the end of the turn. The abrupt change when yaw reaches 180° (to −180°) or −180° (to 180°) does not indicate a turn (24). Flags were used to extract turn metrics (magnitude, duration, and direction). **(B)** The area indicated by the circle in **(A)** shown at higher magnification, with turning patterns as reflected by yaw angle cut into small pieces. The end of the previous turn is the beginning of the following turn, marked by flags (vertical dashed lines). **(C)** Turns including hesitations were identified by the algorithm and defined as one turn when ≥10° angular displacements with identical directions and ≤0.5 s separation occurred.

*Angular displacement around the Z axis* was then plotted as shown in Figure 3A (blue line). This line was then cut into pieces (Figure 3B), where the start of a turn to the right was defined by a change from an increase to a decrease and the end by the change from a decrease to an increase. For the definition of a turn to the left, the situation was defined *vice versa*. The duration (horizontal component of the line) and magnitude (vertical component of the line) of each turn were determined between the start to end of the turn.

Initially, a threshold of 45° for the magnitude of a turn was introduced, and the training data set was analyzed and compared with data obtained from clinical observation. We learned from this analysis that the algorithm split some turns into two turns, when the clinical observers only saw one turn. When analyzing this issue in more detail, we realized that this phenomenon could be explained by subtle hesitations within turns (Figure 3C), which were detected by the algorithm but not by the clinical eye. To adapt the algorithm to the gold standard, a movement with a duration less than 0.5 s and a magnitude less than 10% of the magnitude of the previous and following turns (each must be greater than 10° and have the same direction) was defined as a hesitation, and two turns separated by such a hesitation were defined as one single turn.

#### Turning Analysis

Turn magnitude and duration were extracted using flags from the previous turning detection part (Figure 3A). Turn direction was identified by integration of raw data from the gyroscope. A negative integration value was defined as a left turn and a positive integration as a right turn.

### Statistical Analysis

The degree of agreement for turn detection between the two clinical observers was calculated using interclass correlation. Turns that were differently classified by the clinical observers (LH and ME) regarding presence (present/absent), were discussed posteriorly until consensus was reached, to provide a gold standard data set with perfect agreement for the validation of the algorithm. In case of doubt, a third clinical observer (WM) was involved in the decision process.

Then, data from the training cohort were used to calculate true positive, true negative, false positive, and false negative values for presence of turns based on increasing magnitude thresholds of turns (45°–110°) implemented in the algorithm (comparing with 45° threshold for turns detected by clinical observers). The threshold for automatic detection was selected, taking into account the occurrence of turns detected in the analysis and the agreement to the clinical-observer-determined turns. Inter-rater agreement to detect turns and also the agreement between the clinical observers and the algorithm to identify these episodes was compared with Cohen’s kappa value.

The turns extracted from the test data by the algorithm and the clinical observers were used to calculate sensitivity, specificity, accuracy, positive predictive value, and negative predictive value for turns above the selected threshold. Agreement on time at which turns were detected by algorithm and by the clinical observers was assessed using Bland–Altman plots.

Turn magnitude, duration, and direction were extracted from videos by LH and ME. These results were compared with results yielded by the algorithm using Bland–Altman plots and Chi square. Analyses were performed using JMP 12.2.0 software.

## Results

### Cohorts

Demographic and clinical data of PD patients and controls included in the training and test cohorts, respectively, are presented in Table 1. Cohen’s kappa value between the two clinical observers for the detection of turns in the video tapes was 0.92.

### Algorithm Development Group

A total number of 1,035 turns ≥45° were detected by the algorithm, and 758 by the clinical observers. Comparison between algorithm and clinical observer data, using ascending turn magnitude thresholds in the algorithm, revealed that the most practical result was obtained using a threshold of 90° (Table 2). This threshold was then implemented in the algorithm and used for further analyses.

**Table 2:** **Validation values for the algorithm, based on increasing turn magnitudes, from the training cohort**.

Turning angle	Cohen’s kappa	Accuracy	Sens	Spec	PPV	NPV	True positive turns	False positive turns
≥45°	0.15	0.66	0.92	0.21	0.67	0.59	694	341
≥60°	0.53	0.78	0.91	0.61	0.76	0.82	627	194
≥70°	0.64	0.83	0.91	0.71	0.82	0.85	607	148
≥80°	0.68	0.84	0.90	0.77	0.83	0.87	586	123
≥90°	0.72	0.86	0.90	0.82	0.85	0.88	565	98
≥100°	0.76	0.88	0.90	0.86	0.87	0.89	529	82
≥110°	0.77	0.89	0.89	0.88	0.88	0.90	511	72

*758 turns ≥45° defined by video observation were included*.

*As expected, validation values improved with increasing magnitude of turns, at the expense of number of turns included in the analysis. Based on these values, we decided to use a threshold magnitude of 90° for validation purposes*.

*NPV, negative predictive value; PPV, positive predictive value, Sens, sensitivity; Spec, specificity*.

### Algorithm Validation Group

**Figure 4:**
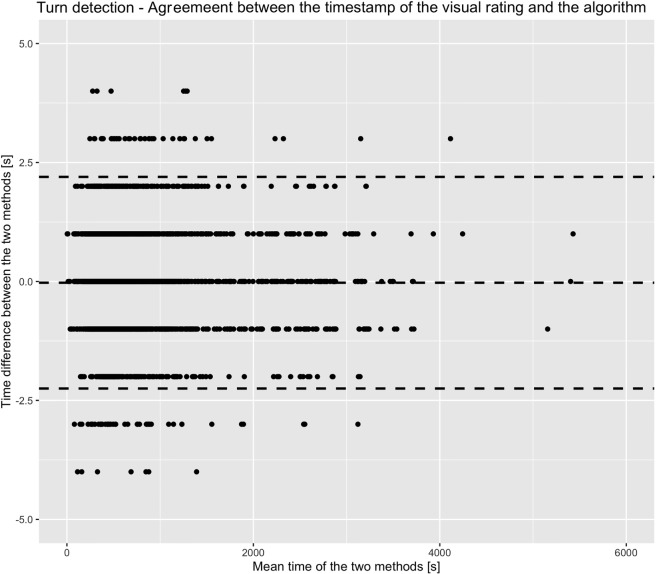
**Bland–Altman plot illustrating the agreement between the time of turn detection from the algorithm and from the clinical observers**. Dashed lines indicate mean and 95% confidence intervals of the difference of observation in seconds. The illustration indicates the high agreement between the two methods (mean turn duration was 2.4 s).

Based on the 90° turn magnitude threshold, the Bland–Altman plots for the detection of a turn showed a mean difference of −0.03 s (SEM, 0.02 s; 95% confidence interval, −2.25 to 2.20 s) for the detection of corresponding turns (Figure 4). Accuracies for detection of turns were 0.91 (overall cohort), 0.92 (PD patients under medication OFF-condition), 0.83 (PD patients under medication ON-condition), and 0.92 (controls). Turn magnitude as calculated by the algorithm was similar to gold standard (155.09° with clinical observers, 155.15° with the algorithm). The mean difference was −0.06° (SEM, 0.14°). Turning duration as calculated by the algorithm was also similar to gold standard (2.44 s with clinical observers, 2.43 s with the algorithm). Turning duration difference between gold standard and algorithm was 0.004 s (SEM, 0.005 s). Turn direction identification reached an accuracy of 0.995. Table 3 presents detailed results about the validation of turn detection and evaluation.

**Table 3:** **Validation values for detection of 90° turns, separated by groups**.

Cohorts	Cohen’s kappa	Acc	Sens	Spec	NPV	PPV	Turns detected by algorithm	Turns detected by clinical observers	True positive turns	False positive turns	True negative turns	False negative turns
All	0.82	0.91	0.93	0.89	0.90	0.92	2,393	2,304	2,150	243	1,884	154
PD ON-med.	0.68	0.83	0.92	0.78	0.74	0.93	681	652	602	79	609	50
PD OFF-med.	0.84	0.92	0.94	0.89	0.92	0.93	934	905	843	91	688	62
Controls	0.84	0.92	0.94	0.89	0.91	0.93	778	747	705	73	587	42

*Acc, accuracy; NPV, negative predictive value; PD, Parkinson’s disease; OFF, medication off state; ON, medication on state; PPV, positive predictive value; Sens, sensitivity; Spec, specificity*.

*Validation values on a sample by sample basis are comparable (not shown)*.

## Discussion

In this study, we present an improved method for the analysis of turns in a real-life-like environment based on 6DOF data from a sensor worn on the lower back, with very high sensitivity, specificity and accuracy, and highly accurate turn metrics, compared against video observation. We focused on turning, as this activity is highly relevant for the proper performance of daily activities: on average, this movement is performed about 1,000 times per day [(8) and own observations in PD patients]. It is associated with dynamic balance capacity (2) and risk of falling (3, 4) in both, older adults and PD patients.

The algorithm reached a Cohen’s kappa value of 0.72 with visual observation. This value reflects a substantial agreement (25) of the algorithm with the gold standard and supports the usefulness of the algorithm for unobserved measurements. However, it does not consider possible errors in activities such as driving and cycling. These potential sources of error need further investigation.

The increase of accuracy with increasing magnitude of a turn (as shown in Table 2) is mainly driven by (shortcomings of) the gold standard and not by algorithm limitations. It is difficult to estimate turn magnitude with naked eyes. This explains why the performance improves with increasing thresholds, as larger turns are more evident. Nevertheless, we feel that a validated algorithm collecting turning information with this magnitude or larger in free-living environments should cover most daily relevant aspects of this movement.

The algorithm experienced relatively low specificity for PD patients under medication ON-condition. When going into the video data, we learned that the algorithm had problems in detecting turns with large radius in these patients. The algorithm split the large turns into several little turns below 45° threshold, which led to more false negative turns. When we increased the hesitation values included in the algorithm, it decreased the sensitivity values, accordingly.

When these issues are sufficiently considered, we believe that the algorithm, which can be applied to almost all currently available sensor systems (i.e., with accelerometer and gyroscope) worn at the lower back, is applicable for home-based assessment of turning in the investigated cohorts. The algorithm for turning detection and analysis presented here can be of particular value, as it also provides highly valid parameters for the estimation of the *quality* of respective turns, such as magnitude, duration, and direction of the turn.

This algorithm has some limitations. First, as illustrated in Figure 3A, the orientation estimates during the periods from 0 to 10, 25 to 30, 43 to 50, and 55 to 60 s, during which the participant was not moving, slightly decrease. The reason is that gyroscopes have small biases, which will be integrated over time and will change yaw. However, this slow tendency has a negligible effect on detection of faster dynamics such as turning. Hence, no further action was taken to eliminate this error, also to keep computational load as low as possible. This yaw integration error could be reduced with a magnetometer (compass), i.e., using a 9DOF sensor. However, we abstained from including magnetometer data in our algorithm, as it could reduce the specificity of results. Magnetometers are influenced by ferromagnetic objects, which are regularly encountered in daily life environments. Another limitation may be the videotaping approach. We followed the study participants with a hand-held camera and missed some turns when they were out of camera range. However, we argue that the number of turns during those periods were only 8% of the total number of turns. A strength of the algorithm is that it takes turn hesitations into consideration, i.e., the algorithm decides, based on an empirically defined threshold, when a no-turning phase is too short to separate one turn from another. This is a relevant addition particularly for PD patients, as hesitations during a turn occur relatively often and would lead to an overestimation of the numbers of turns and to relevant issues when qualitative parameters are extracted from respective turns. Moreover, sensors without magnetometer are readily available and easily accessible.

In conclusion, this study reports about high validity values of an algorithm for the detection and analysis of turns in older adults and PD patients based on 6DOF inertial sensor data worn on the lower back.

## Author Contributions

MP and WM were responsible for the conception and design of the study. ME, TH, LH, DS, DB, and WM contributed to acquisition of data. MP, ME, LH, MH, GF, AS-F, DS, JD, and WM were involved in analysis and interpretation of data. MP and WM drafted the first version of the article. MP, ME, LH, MH, JF, AS-F, DS, JD, and WM revised it critically for important intellectual content. All authors gave final approval of the version to be submitted.

## Conflict of Interest Statement

The authors declare that the research was conducted in the absence of any commercial or financial relationships that could be construed as a potential conflict of interest.
